# A single identified glomerulus in the zebrafish olfactory bulb carries the high-affinity response to death-associated odor cadaverine

**DOI:** 10.1038/srep40892

**Published:** 2017-01-19

**Authors:** Milan Dieris, Gaurav Ahuja, Venkatesh Krishna, Sigrun I. Korsching

**Affiliations:** 1 Institute of Genetics, Biocenter, University at Cologne, Cologne, Germany

## Abstract

The death-associated odor cadaverine, generated by bacteria-mediated decarboxylation of lysine, has been described as the principal activator of a particular olfactory receptor in zebrafish, TAAR13c. Low concentrations of cadaverine activated mainly TAAR13c-expressing olfactory sensory neurons, suggesting TAAR13c as an important element of the neuronal processing pathway linking cadaverine stimulation to a strongly aversive innate behavioral response. Here, we characterized the initial steps of this neuronal pathway. First we identified TAAR13c-expressing cells as ciliated neurons, equivalent to the situation for mammalian *taar* genes, which shows a high degree of conservation despite the large evolutionary distance between teleost fishes and mammals. Next we identified the target area of cadaverine-responsive OSNs in the olfactory bulb. We report that cadaverine dose-dependently activates a group of dorsolateral glomeruli, at the lowest concentration down to a single invariant glomerulus, situated at the medial border of the dorsolateral cluster. This is the first demonstration of a single stereotyped target glomerulus in the fish olfactory system for a non-pheromone odor. A mix of different amines activates many glomeruli within the same dorsolateral cluster, suggesting this area to function as a general amine response region.

Odors drive many behaviors, from food and prey search over predator evasion to many social and reproductive behaviors. Responses to many odors have to be learned, but some odors elicit innate responses. Among the latter are fear responses elicited by predator odors such as trimethylthiazoline, also known as fox odor. Such responses are characterized by freezing and other defensive behaviors in mice (i.e., typical fear responses)^1^. Currently a great deal of effort goes into unraveling the molecular basis and identifying the neuronal circuits for such types of behaviors. In this context the location of the glomeruli activated by such odors is of interest, since olfactory glomeruli form an invariant pattern with fixed positions, resulting in a functional map^2^,^3^,^4^,^5^.

An important receptor class mediating fear and/or aversive responses are the trace amine-associated receptors (TAARs). They comprise a small group of little over a dozen olfactory receptors in mammals^6^. Surprisingly, this receptor class is several-fold larger in teleost fish species, with over one hundred receptors in zebrafish^7^. Recently, one of these receptors, zebrafish TAAR13c, has been identified *in vitro* as a high affinity receptor for cadaverine. *In vivo*, cadaverine activated TAAR13c-expressing neurons, and elicited pronounced aversive behavior^8^. However, it was not known, which type of olfactory sensory neurons (OSNs) expresses TAAR13c, and the neuronal circuits involved in processing cadaverine signals remained elusive beyond the level of the olfactory sensory surface.

Here, we show that TAAR13c is expressed exclusively in ciliated neurons. We identify a single invariant glomerulus in the dorsolateral cluster of glomeruli in the olfactory bulb as high affinity processing unit for cadaverine. We observe increasing recruitment of neighboring glomeruli with successively higher cadaverine concentrations. Furthermore, many glomeruli of the dorsolateral cluster respond to a broad amine mix, suggesting this area as general amine response region, and thus a chemotopic representation of amine odors at the level of the olfactory bulb. These findings provide a first step in the analysis of the neural circuits activated by amines and in particular the death-associated odor cadaverine.

## Results

### Taar13c is expressed in ciliated neurons that are G_olf_-positive

The olfactory epithelium of zebrafish comprises four populations of OSNs^9^. Ciliated neurons express olfactory marker protein (*OMP*), microvillous neurons express transient receptor potential channel C2 (*TRPC2*), and crypt and kappe neurons have been labelled by S100-ir and Go-ir, respectively^4^,^9^,^10^,^11^,^12^. To identify the OSN type expressing TAAR13c, we first performed IHC with α-TAAR13c on olfactory epithelia from transgenic animals expressing fluorescent marker proteins specific to ciliated and microvillous OSNs. In one of these lines, Tg(*OMP:lynRFP*), RFP is expressed under the control of the *OMP* promoter, and labels exclusively ciliated neurons. In the other line, *Tg(TRPC2:gap-Venus)*, VENUS is expressed under the control of the *TRPC2* promotor, i.e. in microvillous receptor neurons^10^.

**Figure 1:**
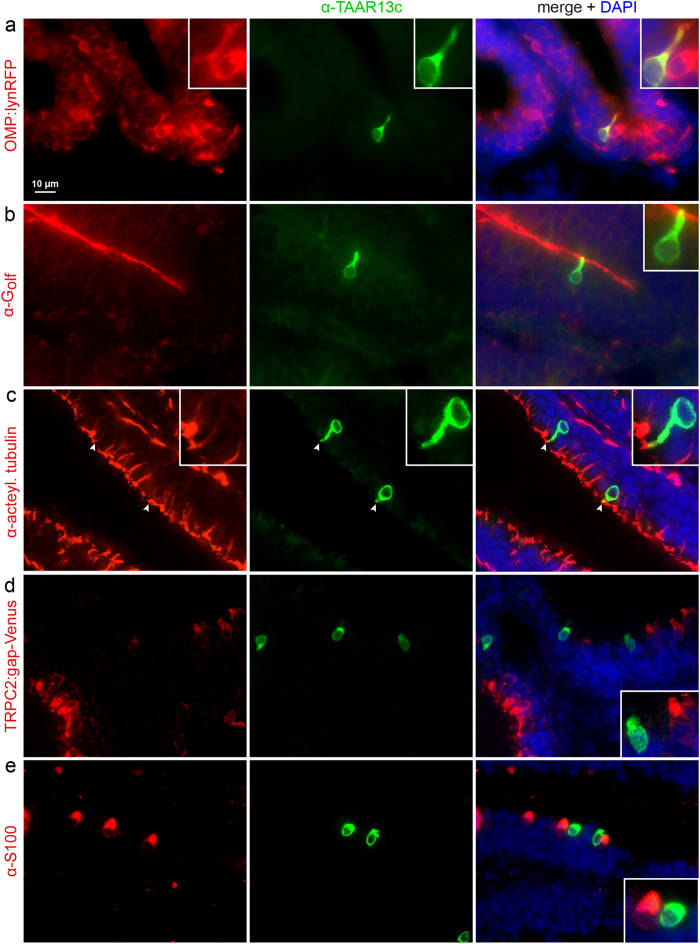
TAAR13c-positive neurons express ciliated markers, but not those of other OSN populations. Horizontal sections of adult zebrafish olfactory epithelium were double-labelled with several cell type markers (left column, red fluorescence) and TAAR13 antibody (middle column, green fluorescence). Right column, merged images shown together with DAPI (blue fluorescence). Insets show single cells at higher magnification. (**a**) TAAR13c-positive cells are also positive for OMP in the *OMP:lynRFP* transgenic line. (**b**) The dendritic knob of TAAR13C-positive neurons is also labelled by G_olf_ antibody, as seen by the yellow color. (**c**) The dendritic knob of TAAR13c-positive neurons is labelled with acetylated tubulin antibody (arrowheads). (**d**) No overlap is seen between TAAR13c antibody staining and TRPC2 expression as visualized in the *TRPC2:gap-Venus* transgenic line (here represented as red for consistency reasons). (**e**) No overlap is seen between TAAR13c and S100 antibody staining.

**Figure 2:**
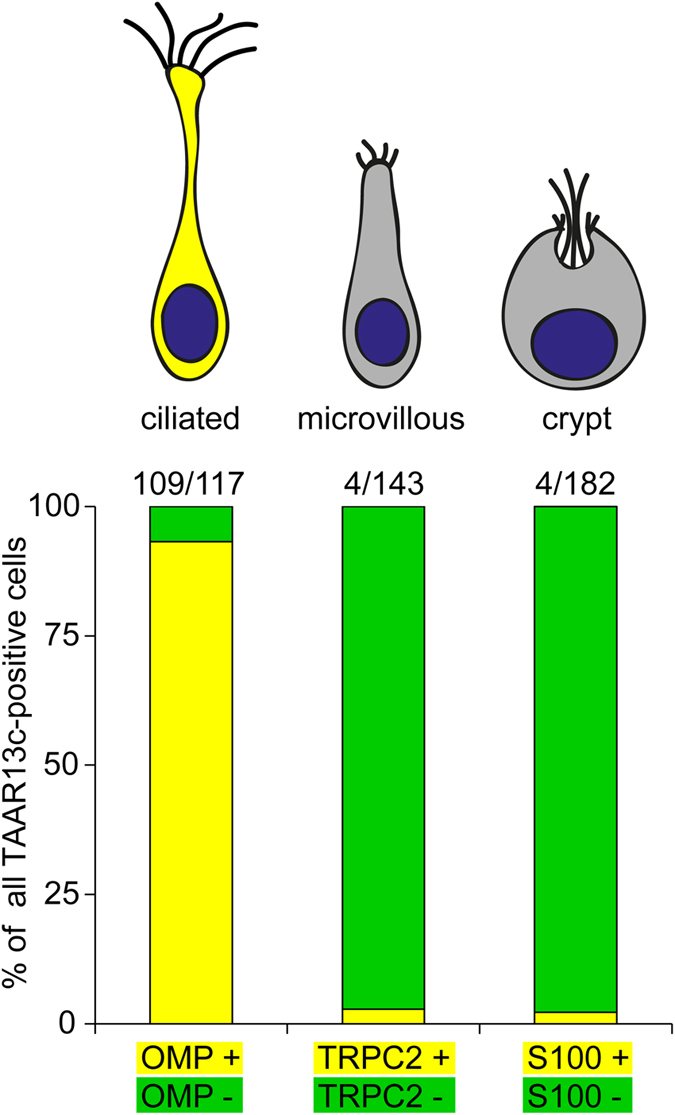
Quantitative evaluation of co-localization of TAAR13c-positive neurons with OSN cell type markers. Labeling was performed as described in Fig. 1, and over one hundred TAAR13c-labelled cells were evaluated for co-localization with several cell type markers. Results are shown as bar graph below the schematic representation of the three cell types examined. Co-localization is indicated by yellow, non-co-localization by green segments. Nearly all TAAR13c-positive cells were also positive for OMP, a ciliated neuron marker. In contrast, almost no co-localization with TAAR13c staining was observed for TRPC2 and S100, markers for microvillous and crypt neurons, respectively.

We observed a very robust colocalization of anti-TAAR13c antibody and RFP fluorescence (>93% colocalization, Figs 1a and 2), suggesting that TAAR13c is expressed in ciliated neurons. Consistent with this observation we did not observe co-labelling of TAAR13c with any of the markers for the other OSN populations. Only very rarely TAAR13c-labelled cells appeared to be Venus-positive or S100-ir-positive (<3% co-localization in each case, see Figs 1d,e and 2). We expect that these rare occurrences reflect the close neighborhood of the respective cells, since the olfactory epithelium is densely packed with neurons^13^. Furthermore these cells also constituted a very small percentage of the Venus-positive or S100-ir-positive cell populations (<3% co-localization in both cases, see Fig. 2). We conclude that TAAR13c is exclusively expressed in ciliated OSNs.

Ciliated neurons express tubulin, which is the main structural element of cilia^14^. We performed double labelling with antibodies against TAAR13c and acetylated tubulin, and observe expression of tubulin at the apical tip of dendrites from TAAR13c-positive OSNs (Fig. 1c). Furthermore ciliated neurons are expected to express G_olf_ as their specialized G alpha protein^15^. We could also show the presence of G_olf_ within these cilia (Fig. 1b). The presence of tubulin and G_olf_ in TAAR13c-positive cells further confirms their nature as ciliated neurons.

### Dose-dependent activation of dorsolateral glomeruli by the TAAR13c ligand cadaverine

**Figure 3:**
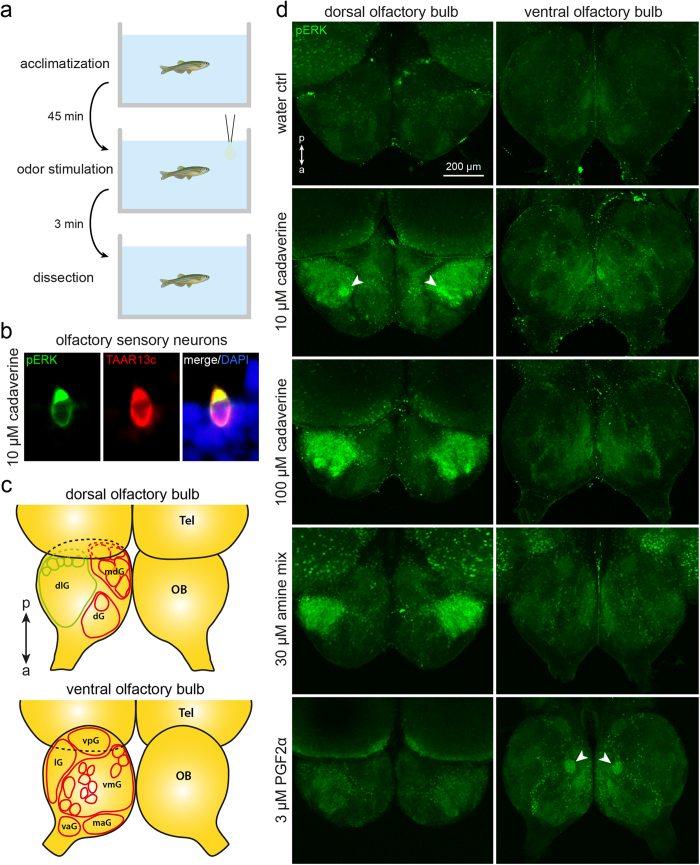
Odor-induced neuronal activation shown by pERK immunohistochemistry in whole mounts of zebrafish olfactory bulb. (**a**) The time course of the experiments is shown schematically. (**b**) Exposure to 10 μM cadaverine elicits robust and specific pERK antibody labelling of TAAR13c-positive neurons, consistent with previous observations^8^. (**c**) A schematic drawing of the olfactory bulb (OB; drawn using main positional information from ref. 4) shows the main glomeruli and glomerular groups (dlg, dorsolateral cluster; dG, dorsal cluster; mdG, mediodorsal cluster; lG, lateral chain; vpG, ventroposterior glomerulus; vmG, ventromedial glomeruli; vaG, ventroanterior glomerulus; maG, medioanterior cluster) visible from the dorsal (top scheme) and ventral (bottom scheme) side of the olfactory bulb; Tel, telencephalon; a↔p, anterio-posterior axis. (**c**) pERK levels (green) are visualized by immunohistochemistry. All pictures show maximum projections from confocal z-stacks of the bilateral olfactory bulb, as seen from dorsal (left column) or ventral (right column). Weak diffuse background fluorescence is present in all assay conditions. Top row, no activated glomeruli were seen in the negative control (water as stimulus). Second row, 10 μM cadaverine activates few glomeruli, one of them strongly (arrowhead). Third row, 100 μM cadaverine activates many glomeruli within the dorsolateral cluster. Fourth row, a mix of amines results in a strong activation restricted to the dorsolateral cluster. Bottom row, 3 μM PGF2α activates a single glomerulus (arrowhead) in the ventral olfactory bulb.

Phosphorylation of ERK (extracellular signal-regulated kinase) has been established as robust signal to measure neuronal activation^16^,^17^. Recently the suitability of this marker for tracking cadaverine responses in the olfactory epithelium of zebrafish both by IHC and Western blot has been demonstrated^8^. We wished to see whether this method could also be used to identify the target glomerulus or glomeruli of cadaverine-sensitive OSNs in the olfactory bulb, using a similar stimulus protocol as described in ref. 8. This would yield an estimate of the number of different olfactory receptors responding to cadaverine, since each glomerulus is formed by the converging axon terminals of OSNs expressing a particular receptor in mouse^2^ as well as zebrafish^12^. Fish were subjected to three minute odor stimulation (Fig. 3a), followed by a whole mount immunohistochemistry protocol for the olfactory bulb. First, we confirmed the presence of cadaverine-sensitive neuronal somata (Fig. 3b, *cf*.^8^) in our assay conditions. Then we proceeded to investigate the activation of glomeruli in the olfactory bulb.

**Figure 4:**
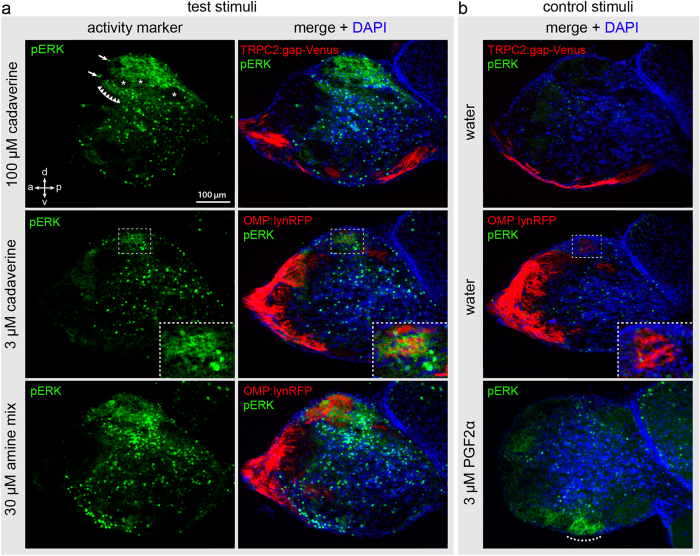
Odor-induced neuronal activation shown by pERK immunohistochemistry on sections of zebrafish olfactory bulb. Adult fish were exposed to odor as shown in Fig. 3 and cryostat sections of the olfactory bulb were processed for immunohistochemical detection of pERK. The sections with maximal pERK signal for the respective odor are shown. (**a**) Top row, exposure to high level of cadaverine (100 μM); middle row, exposure to low level of cadaverine (3 μM); bottom row, exposure to a mix of 13 different amines (30 μM). Left column of panel (a) shows the pERK signal, right column shows the pERK signal merged with DAPI and the cell type markers TRPC2 and OMP as indicated on the panels. Note a strong signal in dorsal glomeruli for high cadaverine and the amine mix, and a much more restricted dorsal signal for the low cadaverine concentration. (**b**) Stimulation with water as negative and PGF2α as positive control as indicated. No pERK labelling is visible in the water control in both the *TRPC2:gap-Venus* and *OMP:lynRFP* transgenic lines (both shown as red fluorescence for consistency reasons). PGF2α activates a ventral glomerulus (dotted line), as described in ref. 18. Arrows indicate exemplary pERK-labelled cell bodies in the OB. Row of triangles shows a weak labelling of the neuropil in the vicinity of the dlG. Asterisks point out pERK-negative glomeruli interspersed with the active glomeruli in the dlG. Dashed boxes in the middle row show enlarged views of the single-cadaverine responsive glomerulus, which is pERK-positive after stimulation with 3 μM cadaverine and pERK-negative in the negative control.

Stimulation with 100 μM cadaverine evoked a strong pERK signal in the dorsolateral cluster of glomeruli (dlG, nomenclature according to Braubach *et al*.^4^), see Fig. 3c,d. This signal covered a considerable area of the olfactory bulb, corresponding to the whole dorsolateral cluster, i.e. exceeding the size of even the biggest glomerulus severalfold (Fig. 3d). Thus, at these high concentrations many glomeruli and therefore olfactory receptors appear to carry the cadaverine response. No pERK signals were seen in the dorsomedial cluster (mdG) and more anterior regions. Although the dorsolateral cluster appeared to be rather homogenously labelled in the whole mount analysis, in sections it became clear that cadaverine-responsive and -unresponsive glomeruli intermingle within this cluster (Fig. 4a).

When using lower cadaverine concentrations, the signal became more spatially restricted. After stimulation with 3–10 μM cadaverine only a single to very few glomeruli at the medial boundary of the dlG were labelled by α-pERK antibody (Fig. 3d, see also SI Fig. 2). No signals were observed in the dlG in the negative control, stimulation with water (Fig. 3d). Also, PGF2α, a pheromone that is known to be processed by a specific glomerulus in the ventromedial cluster (vmG)^18^, did not evoke any responses in the dlG, but did elicit a pERK signal in the reported ventral glomerulus (Fig. 3d). Together, these results show the specificity of the cadaverine-induced pERK signal and are consistent with the possibility that at low cadaverine concentrations a single glomerulus is activated. pERK signals in whole mount olfactory bulbs became very dense and it was correspondingly difficult to distinguish individual activated glomeruli with increasing cadaverine concentrations. Thus, a thorough investigation of cadaverine-responsive glomeruli position and number, including an unequivocal identification of individual glomeruli activated by cadaverine required immunohistochemical detection of pERK on cryostat sections of the olfactory bulb (see below).

### A single glomerulus with stereotyped position is activated by low concentrations of cadaverine

Fish were subjected to different odor stimuli for 3 minutes, as before, and sacrificed. Olfactory bulbs were dissected, and cryostat sections were used for immunohistochemical experiments. We used wildtype fish as well as transgenic reporter lines expressing fluorescent proteins in either ciliated or microvillous OSNs, Tg(OMP:lynRFP) and Tg(*TRPC2:gap-Venus*), respectively. Immunohistochemical results were undistinguishable between wildtype fish and reporter lines. Cadaverine concentrations ranged between 3 μM and 100 μM. Glomeruli were identified by morphology and either by the presence of synaptic vesicle protein 2, an accepted synaptic marker^19^, or by the absence of nuclei stained by DAPI.

**Figure 5:**
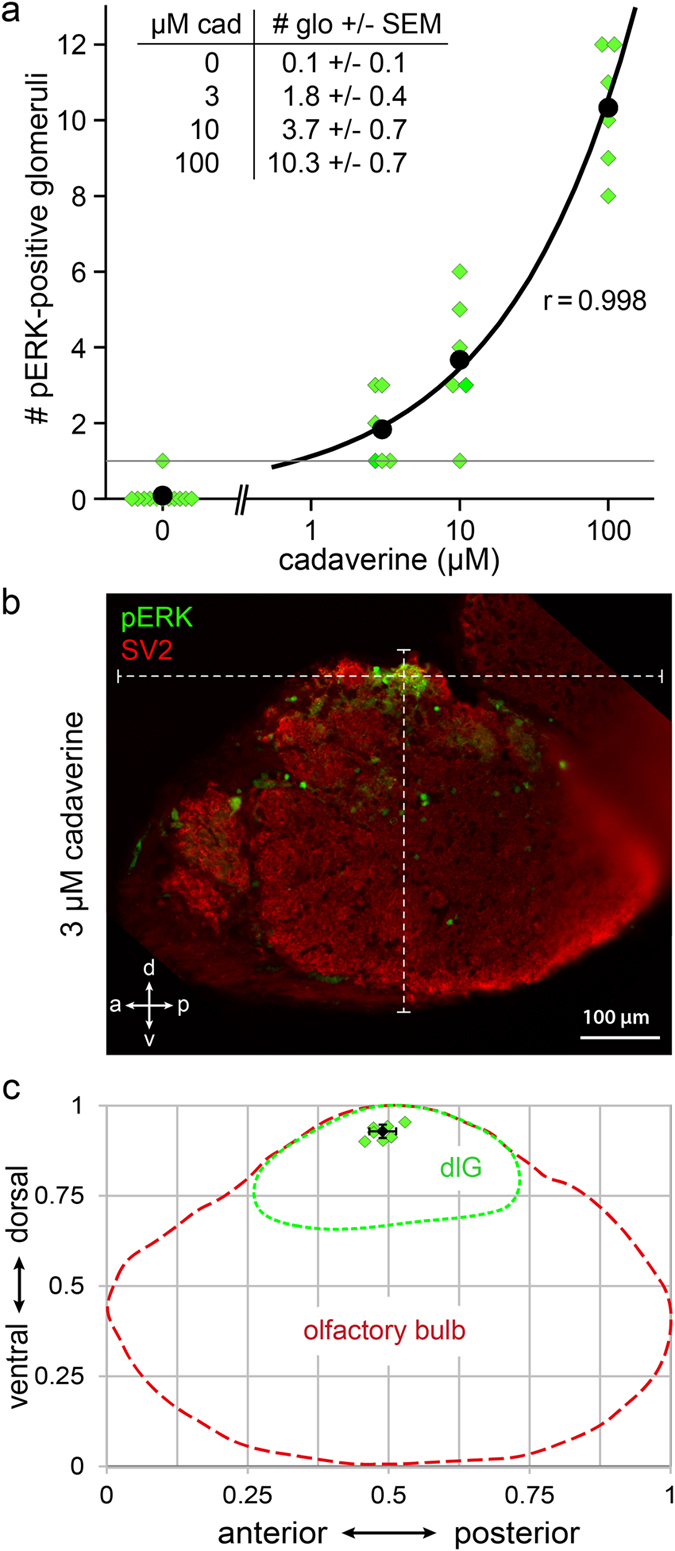
A single invariant glomerulus is activated by exposure to low concentrations of cadaverine. (**a**) Number of glomeruli activated in response to different doses of cadaverine was counted from sections. Water did not evoke any pERK signal in the dorsolateral cluster in all but one cases (n = 12). At 3 and 10 μM cadaverine one to few glomeruli were labelled (see table inset). At 100 μM cadaverine about 10 glomeruli were labelled (n = 6 for each cadaverine concentration used). (**b**) Exemplary image of a single glomerulus labelled by pERK, double labelling with SV2 to visualize all glomeruli in this section. White dashed lines intersect in the glomerulus center and show overall a↔p and v↔d length of the olfactory bulb section used for calculating the normalized glomerulus position. (**c**) Schematic representation of the dlG_cad_ coordinates measured in the olfactory bulb. Red and green dashed lines represent olfactory bulb and dorsolateral cluster contours, respectively. Green squares represent single measurements from 7 different bulbi. Black square and error bars represent mean value ± SD.

At 100 μM cadaverine 10.3 ± 0.7 (mean ± SEM; n = 6) glomeruli of the dorsolateral cluster showed pERK signals (Figs 4a and 5a). These glomeruli were distributed across the dorsolateral cluster, interspersed with non-responding glomeruli (Fig. 4a). Other regions of the olfactory bulb did not contain any reproducible cadaverine-response. A tenfold lower concentration of cadaverine, 10 μM, activated only a few dorsolateral glomeruli (3.7 ± 0.7, mean ± SEM; n = 6), in one case only a single glomerulus. At the lowest concentration tested, 3 μM, a further decrease in number of labelled glomeruli was seen (1.8 ± 0.4, mean ± SEM; n = 6), and in three of these cases only a single glomerulus was labelled (Figs 4a and 5a). Giving water as a control stimulus did not evoke a pERK signal in the cadaverine-sensitive glomeruli. In fact, no dlG glomeruli were labelled in the absence of cadaverine in all but one case (n = 12). We suspect that such rare exceptions are caused by minor contaminants present in the water.

We were interested to see, whether the single glomerulus labelled might be invariant, i.e. constitute the same stereotyped glomerulus, and determined its position in the olfactory bulb. The position of this single labelled glomerulus was strikingly invariant (Fig. 5b,c), with coordinates a↔p = 0.49 ± 0.02 and v↔d = 0.93 ± 0.02 (mean ± SD; n = 7). In the third dimension, medial to lateral (m↔l), we determined the glomerulus position from whole mount samples treated with low cadaverine concentrations and found it to be located at m↔l coordinate 0,57 ± 0,03 (mean ± SD; n = 5).

This extremely high degree of precision in the three-dimensional location observed in four independent experiments suggests that all occurrences of a single labelled glomerulus indeed constitute the same stereotyped glomerulus, hereafter called dlG_cad_, which is robustly activated by stimulation with cadaverine at 3 and 10 μM concentrations. This glomerulus was also recognizable by position in those experiments, where small groups of glomeruli were labelled (SI Fig. 1). Even directly adjacent glomeruli exhibited clearly distinguishable coordinates (SI Fig. 1). Taken together, dlG_cad_ appears to be innervated by OSNs expressing the most sensitive cadaverine receptor still able to elicit a pERK signal at 3 μM cadaverine. At higher concentrations of cadaverine, successively more glomeruli and thus olfactory receptors were recruited to the cadaverine response (Fig. 5a).

The dlG_cad_ glomerulus is formed by axons of ciliated OSNs, as confirmed by IHC with α-pERK antibody on the transgenic reporter line expressing RFP under control of the *OMP* promoter (see Fig. 4a). Microvillous OSNs, which mainly project into the ventral part of the olfactory bulb, do not play a role in the processing of cadaverine stimuli, because even at 100 μM concentration cadaverine did not evoke any activity-related pERK signal in the glomeruli formed by microvillous OSNs (Fig. 4a).

We often observed bilateral symmetry not only for the position of the dlG_cad_ glomerulus, but also for other glomeruli in those experiments, where few glomeruli were activated and thus easily distinguishable, see e.g. Fig. 3d. Furthermore, the number of activated glomeruli was nearly identical for the left and right olfactory bulb of an individual fish (SI Fig. 2). Thus a stereotyped subset of glomeruli might be activated at each concentration of cadaverine, according to the respective affinities of the underlying receptors to cadaverine.

### pERK may constitute a general marker of neuronal activation both for OSN stimulated by other odorants and for secondary neurons

In all specimens we found pERK-labelled cell bodies in the vicinity of activated glomeruli and a weak pERK labelling of neuropil below the activated glomeruli (Fig. 4). These signals might correspond to somata and dendrites of mitral cells innervating the activated glomeruli and involved in the downstream processing of the cadaverine stimulus. Such signals were substantially weaker in negative controls, and are not to be confused with sparse labelled cell bodies distributed all over the olfactory bulb, which did occur in negative controls and cadaverine-exposed animals likewise (Fig. 4).

As further control we used stimulation with PGF2α, since the glomerulus activated by PGF2α, a large ventromedial glomerulus, is already known^18^,^20^. We confirmed the activation of a large ventromedial glomerulus by IHC with α-pERK antibody on cryostat sections, whereas the dorsolateral cluster was not activated by this pheromone (Fig. 4b). PGF2α-sensitive OSNs were previously shown to play a role in male courtship behavior^20^.

### The dorsolateral cluster appears to be dedicated to processing of amine odors

The activation of large parts of the dorsolateral cluster at high concentrations of cadaverine led us to hypothesize that this cluster might be dedicated to detection of amine odors in general. To examine this assumption we have applied a mix of thirteen amines chosen to be representative of many different amine classes^21^. This mix contains primary, secondary and tertiary amines, as well as aliphatic and aromatic amines. Exposure to the amine mix resulted in intense activation of the dorsolateral cluster. Outside of this cluster, e.g. in dorsomedial and more anterior regions, and generally ventral, no glomeruli were labelled (Figs 3 and 4a). pERK labelling performed on tissue sections showed many labelled glomeruli in the dlG, intermingled with some unresponsive glomeruli (Fig. 4a). Current knowledge does not allow to decide, whether the unresponsive glomeruli have non-amine ligands, or whether, perhaps more likely, they might respond to amines structurally different from those examined.

It is not clear whether the amine-responsive glomeruli are restricted to ciliated neurons, as they included *OMP:lynRFP-*positive as well as *OMP:lynRFP-*negative glomeruli (Fig. 4a). There are three OMP-negative OSN populations known in fish, two of which, crypt and kappe neurons, project to the mediodorsal cluster (mdG)^9^,^12^, which was not labelled in response to amine stimuli. This leaves microvillous OSNs as potential innervation of the *OMP:lynRFP*-negative amine-responsive glomeruli of the dorsolateral cluster. Alternatively, the OMP line used may not have complete penetrance for ciliated neurons. In each case, amine responses appear to be limited to the dlG cluster, a strikingly distinct example of chemotopy in the odor responses within the zebrafish olfactory bulb. We would like to note that this result validates pERK as suitable marker for odor-induced neuronal activation also for amines as a novel odorant class in fish olfaction.

## Discussion

Cadaverine is a diamine that is released during bacteria-mediated decay processes^22^,^23^ and thus constitutes a death-associated odor. Previously, cadaverine has been described as the principal activator of zebrafish TAAR13c in decayed fish extract^8^. The ligand spectrum of TAAR13c has been thoroughly investigated, and cadaverine was found to be the optimal ligand^8^. Low concentrations of cadaverine activated mainly TAAR13c-expressing neurons within the olfactory epithelium, suggesting TAAR13c as important element of the neuronal processing pathway that leads from cadaverine stimulation to a strongly aversive behavioral response in zebrafish^8^.

Here we set out to characterize the initial steps of the neuronal pathway leading from cadaverine sensation to the initiation of an innate behavior. First we identified the cell type of TAAR13c-expressing cells as ciliated neurons using several markers for ciliated, microvillous, and crypt neurons^4^,^10^,^11^. Mammalian *taar* genes likewise have been reported to be expressed in ciliated neurons^6^,^24^. Thus the expression of *taar* genes in ciliated neurons is conserved between fishes and mammals despite their large evolutionary distance.

Next we identified the target area of cadaverine-responsive OSNs in the olfactory bulb, the first station in the brain to process olfactory information. We report that cadaverine dose-dependently activates a group of dorsolateral glomeruli, down to a single invariant glomerulus at the lowest concentration, situated at the medial border of the dorsolateral cluster. Because this glomerulus is a member of the dense dorsolateral cluster of about 50 glomeruli with rather homogenous morphology, it is not distinguishable by morphological criteria alone. However, the extremely high reproducibility of its position in normalized coordinates leads us to strongly suggest that this is indeed an invariant glomerulus.

Our results constitute the first demonstration of a single stereotyped target glomerulus in the fish olfactory system for a non-pheromone odor. In the mammalian olfactory system a similar situation has been described for musk odor: at higher concentrations small groups of glomeruli respond, but at very low concentrations a single stereotyped glomerulus remains active^25^.

Cadaverine is known to be a stimulus for mammalian OSNs^26^, but so far no dedicated olfactory receptor or target glomerulus for cadaverine has been identified in mammals. The mouse TAAR4 glomerulus has been shown to respond to cadaverine, but sensitivity to its preferred ligand, phenylethylamine, is several orders of magnitude higher, so the physiological role of this glomerulus for cadaverine detection is not clear^24^.

It appears likely that the single invariant cadaverine glomerulus we describe here is innervated by TAAR13c-expressing OSNs, since at low cadaverine concentrations the large majority of activated OSNs do express TAAR13c^8^. Despite repeated attempts we were not able to identify the TAAR13c glomerulus by direct staining with α-TAAR13c antibody, possibly due to the lower number of converging axons (about 100 for TAAR13c, *cf*.^8^) compared to several thousand for rodent glomeruli^27^.

The higher concentrations of cadaverine recruit many glomeruli and therefore presumably other receptors besides TAAR13c. This is consistent with previous investigations showing a large number of activated OSN somata in the olfactory epithelium after stimulation with 100 μM cadaverine that exceeded the number of TAAR13c-expressing cells by far^8^. Thus, the olfactory representation of cadaverine would change with concentration, not only quantitatively but qualitatively, as an increased set of target glomeruli is recruited, *cf*.^28^,^29^. Cadaverine concentrations in the vicinity of a decaying fish may be expected to be in the low micromolar range (source concentration could be in the millimolar range^8^). This is consistent with one or a few target glomeruli being activated.

When an amine mix was used as stimulus, many glomeruli in the dorsolateral cluster were activated, suggesting that this cluster may be dedicated to processing of amine odors. Since the estimated number of glomeruli in this cluster amounts to about 50 glomeruli^3^,^4^, it is conceivable that this cluster may constitute the main and possibly only target region of TAAR receptors. About one hundred different *taar* genes were identified in the zebrafish genome^7^, which would roughly fit to the number of glomeruli in the dorsolateral cluster. It is conceivable that closely related TAAR receptors might be co-expressed in the same cells (*cf*.^30^), which would reduce the number of glomeruli required to express all TAAR family members. Alternatively it cannot be excluded, that the number of glomeruli distinguishable by morphology is an underestimate. A recent publication^31^ suggested that all three classes of zebrafish TAAR receptors^7^ might respond to amines, which would be consistent with the dorsolateral cluster constituting both the amine response region and the target area of TAAR-expressing OSNs. Genetic labelling experiments for ciliated and microvillous neurons show the dorsolateral cluster as a target region of ciliated OSNs^10^, suggesting that amine responses in general are carried by ciliated OSNs.

Interestingly the dorsal position of the amine-responsive region in the zebrafish olfactory bulb corresponds to the dorsal position of the mammalian target region of TAAR-expressing OSNs^32^,^33^. Since many mammalian TAARs have been found to recognize amines^24^,^32^,^34^ this constitutes a remarkable conservation of chemotopy across the evolutionary divide between tetrapods and teleosts.

Even the behavioral relevance of amines as aversive and/or fear signals appears to be conserved between teleosts^8^ and mammals^35^. Thus TAAR receptors and their associated target region in the olfactory bulb appear to constitute an ancient olfactory subsystem dedicated to processing of aversive amine odors. Our findings present a first step in mapping this subsystem in teleosts and provide an excellent stepping stone to analyze the further neuronal processing of an innate aversive behavior in zebrafish, a vertebrate model organism.

## Methods

### Antibodies, tissue and animal handling

Primary antibodies used: α-TAAR13c (rabbit IgG, ployclonal, for source see ref. 8), α-tubulin (mouse IgG, monoclonal; #G7121, Promega), α-G_olf_ (mouse, monoclonal IgG, #sc-55546, Santa Cruz), α-pERK (rabbit IgG, polyclonal; #9101, Cell Signaling), α-pERK (mouse, monoclonal IgG, #9106, Cell Signaling), α-SV 2 (mouse IgG, monoclonal; Developmental Studies Hybridoma Bank, University of Iowa, Iowa City, IA), α-S100 (rabbit IgG; #Z0311, Dako). Note that polyclonal and monoclonal pERK antibodies resulted in very similar staining patterns, with somewhat higher signal/noise ratios for the polyclonal antibody, which was used for all experiments except double-labeling with anti-TAAR13c antibody (results shown in Fig. 3b).

Secondary antibodies used: goat anti-rabbit IgG conjugated to Alexa Fluor 488 (#A21206, Invitrogen), goat anti-rabbit IgG conjugated to Alexa Fluor 594 (#A11012, Invitrogen) and goat anti-mouse conjugated to Alexa Fluor 594 (#A11005, Invitrogen).

Zebrafish strains were kept at 28 °C on 14 h/10 h light/dark cycle and fed with flake food and living *Artemia* twice a day. Wildtype zebrafish (Ab/Tü) as well as transgenic zebrafish lines for ciliated neurons, Tg(*OMP:lyn-mRFP-S*), and microvillous neurons, Tg(*TRPC2:gap-Venus*), were used in this study^10^. Note that OMP used in the transgenic line corresponds to OMP1^36^, with gene name *ompb*. For sections fluorescence was analyzed using a BZ-9000 wide field fluorescence microscope (Keyence, Japan). Whole mount samples were imaged with a LSM 510Meta (Zeiss, Germany).

Animal handling was approved by the governmental animal care and use office (Landesamt für Natur, Umwelt und Verbraucherschutz Nordrhein-Westfalen, Recklinghausen, Germany, Protocol No. 8.87-51.05.20.10.217) and was in accordance with the German Animal Welfare Act as well as with the General Administrative Directive for the Execution of the Protection of Animals Act.

### Stimulation of zebrafish with different odors

For α-pERK immuohistochemistry experiments adult fish between 4 and 9 month of age were used. One night prior to the experiment fish were separated and kept in an individual freshwater-supplied tank. On the next morning fish were brought to a behavior room and allowed to acclimatize for 45 min, before putting them into smaller stimulation tanks and letting them rest for another 45 min. Stimuli were given through a flexible pipe to a final concentration between 1 μM and 100 μM.

The volume ratio was 1:100, i.e. initially the fish might encounter for very short times a stimulus concentration several fold higher than the final value. Larger stimuli volumes would have resulted in less initial deviation from the final concentration, but were found to introduce too much background pERK activation, possibly due to the unavoidable mechanical stimulation by a larger volume flow. The volume ratio chosen represented an empirically determined balance between initial concentration accuracy and noise level. The ensuing variability in initial concentration may explain the variability in the number of activated glomeruli observed at any particular concentration of stimulus.

Cadaverine was purchased from Sigma (#D22606). Amine mix was produced as reported previously^21^. System water served as a negative control stimulus. It was made sure, that the fish were in a calm state at the time point of stimulation and that the experimenter was not visible to the fish. 3 min after stimulation the fish were swiftly immobilized by immersion in ice water and killed by decapitation.

### Immunohistochemistry on cryosections

After stimulation, the heads were fixed in 4% PFA for 1 h before dissection of the OEs and brains. After several washing steps in PBS the OEs were embedded in TissueTek embedding medium (Sakura) and frozen. The brains were fixed in 2% PFA for another 4 h and put in 30% sucrose in PBS overnight. Then, brains were oriented in TissueTek and frozen. 17–20 μm cryosections of the olfactory bulb and 10 μm cryosections of the OE were produced with a cryostat (Leica CM1900) and dried. After rehydration in PBS and blocking in 3% BSA-PBST, sections were incubated over night with a dilution of primary antibody in blocking solution. Primary antibody dilution was 1:250 for all antibodies, except anti-acetylated tubulin (1:1000). On the next day, sections were washed three times in PBS, followed by incubation with secondary antibody (1:250 dilutions in PBST) for 3 h at room temperature. Sections were washed and then mounted with VECTASHIELD^®^ mounting medium (Vector Laboratories) containing DAPI.

### Quantitative evaluation of OSNs and glomeruli

To count co-labelled OSNs in the OE we took high magnification images of corresponding lamellae using at least a 40x objective and looked for fluorescent signal of the OSN-type markers (OMP, TRPC2, S100) in OSNs labelled by α-TAAR13c antibody.

Counts of activated glomeruli were performed on sections to obtain a higher accuracy. Images of serial OB sections were analyzed by counting the pERK-positive glomeruli. Glomeruli were identified by their typical shape, absence of nuclei (DAPI) and/or co-labeling with axons expressing fluorescent proteins in OBs of the transgenic reporter lines used. Particular care was taken to avoid double counting of glomeruli in adjacent sections. In rare cases the distinction of adjacent active glomeruli was not unambiguously possible for the highest concentration of stimuli investigated.

The position of an activated glomerulus was determined by measuring length (anterior-to-posterior, a↔p) and height (ventral-to-dorsal, v↔d) coordinates for its center. These values were then normalized to the overall length and height of the respective bulb section.

### Whole mount immunohistochemistry on olfactory bulb

After decapitation, the dorsal cranium was removed and heads were fixed in 4% PFA overnight. Brains were washed several times in PBST and transferred to −20 °C methanol for ≥4 h or overnight. Brains were then rehydrated in a dilution series of 75%, 50%, 25% methanol in PBST (5 min each) and washed again in PBST. Blocking was performed with blocking solution containing 10% calf serum/1% DMSO in PBST for 1 h. Samples were incubated with primary antibody α-pERK (1:50 in blocking solution) at 4 °C for 5–7 days on a vertical rotator (~12 rpm). After washing several times with PBST, brains were incubated with secondary antibody (1:200 in blocking solution) at 4 °C for ≥3 days. Brains were washed several times in PBST and subjected to tissue clearing using a fructose-gradient (modified from ref. 37). In short, brains were successively incubated in 20%, 40%, 60%, 80%, and 100% fructose in PBS (w/v) on a vertical rotator at RT for 4 h or at 4 °C overnight for each step.

## Additional Information

**How to cite this article**: Dieris, M. *et al*. A single identified glomerulus in the zebrafish olfactory bulb carries the high-affinity response to death-associated odor cadaverine. *Sci. Rep.* **7**, 40892; doi: 10.1038/srep40892 (2017).

**Publisher's note:** Springer Nature remains neutral with regard to jurisdictional claims in published maps and institutional affiliations.

## Supplementary Material

Supplementary Information
