# Supplementary material for: A single identified glomerulus in the zebrafish olfactory bulb carries the high-affinity response to death-associated odor cadaverine
Source: Sci Rep. 2017 Jan 19;7:40892. doi: 10.1038/srep40892 (PMC5244376; doi:10.1038/srep40892)
Supplement: Supplementary Information [file srep40892-s1.pdf]

Supplementary information

A single identified glomerulus in the zebrafish olfactory bulb carries the high-affinity response to death-associated odor cadaverine

Milan Dieris[1], Gaurav Ahuja[1,2,*], Venkatesh Krishna[1,*], Sigrun Korsching[1]

[1] Institute of Genetics, Biocenter, University at Cologne, Cologne, Germany

[2] Current address: Laboratory for Developmental and Regenerative RNA biology, Center for Molecular Medicine Cologne (ZMMK), CECAD - Cluster of Excellence, CECAD Research Center, Max Planck Institute for Biology of Ageing and Institute for Neurophysiology, University of Cologne, 50931, Cologne, Germany.

* These authors contributed equally to this work.

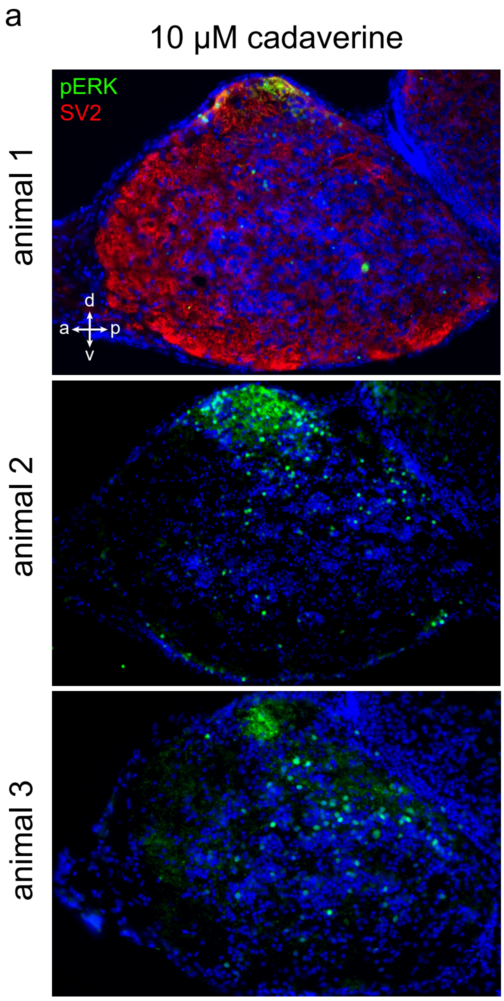

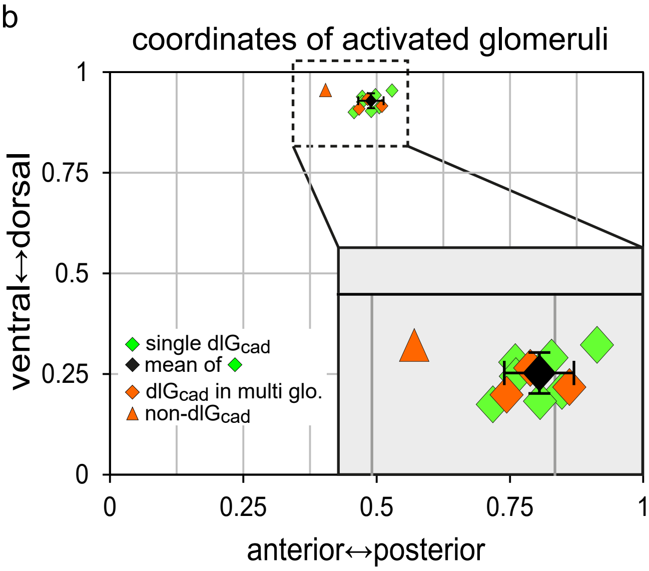

		a ↔ p	v ↔ d
	◆ mean ± SD	0.49 ± 0.02	0.93 ± 0.02
dlGcad	◆ animal 1	0.51	0.92
	◆ animal 2	0.48	0.93
	◆ animal 3	0.47	0.91
	▲ non-dlGcad animal 2	0.40	0.96

SI Figure 1 The dlG$_{cad}$ can also be found in bulbi with multiple pERK-labelled glomeruli

a) α-pERK labels active glomeruli in cryostat sections of the olfactory bulb from 3 different animals stimulated with 10 µM cadaverine. b) Top panel, mapped glomerular positions are shown in the coordinate system used. Green squares, glomerular position, when only a single glomerulus was labelled (n=7, from 3 animals exposed to 3 µM and 1 animal exposed to 10 µM cadaverine, respectively); black square and error bars, average position ± SD; orange squares, three examples for dlG$_{cad}$ positions in cases where multiple glomeruli were labelled; orange triangle, an adjacent non-dlG$_{cad}$ glomerulus. Note the unambiguous identification of the dlG$_{cad}$ glomerulus in all three cases with multiple labelled glomeruli, and the clear distinction even to the directly adjacent non-dlG$_{cad}$ glomerulus (this was the only case in which a non-dlG$_{cad}$ glomerulus was situated in the same section as the dlG$_{cad}$ glomerulus). Bottom panel, numeric values for coordinates of dlG$_{cad}$ and non-dlG$_{cad}$ glomeruli in experiments with multiple glomeruli labelled (orange symbols). For comparison the mean value for the 7 cases, in which a single glomerulus was labelled, is shown (black square). Note the clear distinction between positions of dlG$_{cad}$ and non-dlG$_{cad}$ glomeruli.

a

cad (µM)	animal	number of dlG glomeruli: left OB	number of dlG glomeruli: right OB
	1	0	0
0	2	0	0
	3	0	0
	1	1-2	1
3	2	1	1-2
	3	3	3
	4	1	2
	1	3	3
10	2	n.d.	n.d.
	3	n.d.	n.d.
	1	n.d.	n.d.
100	2	n.d.	n.d.
	3	n.d.	n.d.
	4	n.d.	n.d.

b

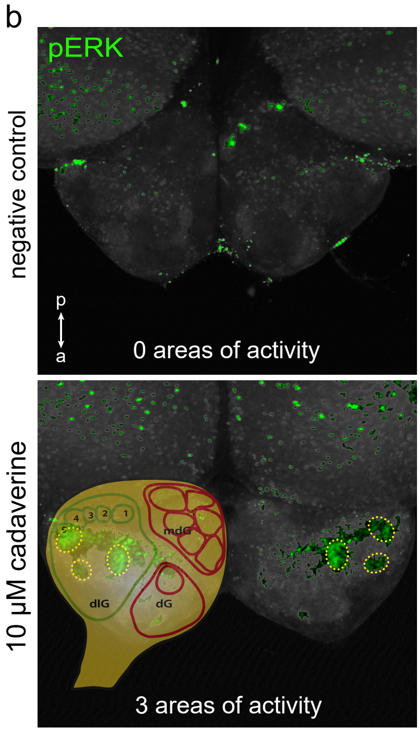

SI Figure 2 Pronounced left-right symmetry of activated glomeruli

In whole mount experiments we compared number and position of pERK-labelled glomerular-shaped areas in the left and right olfactory bulb. a) Table with numbers of activated glomeruli. In all cases, where delineation of glomeruli was unambiguously possible (all experiments with 3 µM, and one with 10 µM cadaverine), we observed a (nearly) equal number of glomeruli labelled on each side. b) Images show pERK-labelling in negative control (top panel) and after exposure to 10 µM cadaverine (bottom panel). Olfactory bulbs are shown from the dorsal view. Activated glomeruli are outlined by dashed circles both in left and right olfactory bulb. A schematic glomerular map (drawn using main positional information from[4]) was made partially transparent and placed over the left olfactory bulb to visualize the position of cadaverine-responsive glomeruli in the dorsolateral cluster. n.d., not determined: an unambiguous identification of single glomeruli was impossible due to high signal density in the whole mount preparations.
